# ER stress preconditioning ameliorates liver damage after hemorrhagic shock and reperfusion

**DOI:** 10.3892/etm.2021.9679

**Published:** 2021-01-22

**Authors:** David Peter Obert, Alexander Karl Wolpert, Nathan Lewis Grimm, Sebastian Korff

**Affiliations:** 1 Department of Anesthesiology and Intensive Care, School of Medicine, Technical University of Munich, 81675 Munich, Germany; 2 Department of Trauma Surgery, University of Heidelberg, 69118 Heidelberg, Germany; 3 Department of Trauma Surgery, Paracelsus Medical University, 90471 Nuremberg, Germany; 4 Department of Orthopaedic Surgery, Duke University Medical Center, Durham, NC 27708, USA; 5 Department of Orthopaedic Surgery, Klinikum rechts der Isar, Technical University of Munich, 81675 Munich, Germany

**Keywords:** hemorrhage, unfolded protein response, tunicamycin, ischemia-reperfusion injury, binding immunoglobulin protein

## Abstract

The mismatch of oxygen supply and demand during hemorrhagic shock disturbs endoplasmic reticulum (ER) homeostasis. The resulting accumulation of unfolded proteins in the ER lumen, which is a condition that is defined as ER stress, triggers the unfolded protein response (UPR). Since the UPR influences the extent of organ damage following hemorrhagic shock/reperfusion (HS/R) and mediates the protective effects of stress preconditioning before ischemia-reperfusion injury, the current study investigated the mechanisms of ER stress preconditioning and its impact on post-hemorrhagic liver damage. Male C56BL/6-mice were injected intraperitoneally with the ER stress inductor tunicamycin (TM) or its drug vehicle 48 h prior to being subjected to a 90 min pressure-controlled hemorrhagic shock (30±5 mmHg). A period of 14 h after hemorrhagic shock induction, mice were sacrificed. Hepatocellular damage was quantified by analyzing hepatic transaminases and hematoxylin-eosin stained liver tissue sections. Additionally, the topographic expression patterns of the ER stress marker binding immunoglobulin protein (BiP), UPR signaling pathways, and the autophagy marker Beclin1 were evaluated. TM injection significantly increased BiP expression and modified the topographic expression patterns of the UPR signaling proteins. In addition, immunohistochemical analysis of Beclin1 revealed an increased pericentral staining intensity following TM pretreatment. The histologic analysis of hepatocellular damage demonstrated a significant reduction in cell death areas in HS/R+TM (P=0.024). ER stress preconditioning influences the UPR and alleviates post-hemorrhagic liver damage. The beneficial effects were, at least partially, mediated by the upregulation of BiP and autophagy induction. These results underscore the importance of the UPR in the context of HS/R and may help identify novel therapeutic targets.

## Introduction

Trauma is the most common cause of death for all age groups below the age of 44 and the single largest cause for years of life lost in the United States (1-3). Acute trauma care, therefore, is not only of the utmost importance from a clinical point of view but also from a public health perspective (4). One of the most dire consequences of severe trauma, and a leading cause of post-injury death, is hemorrhagic shock as it may result in ischemia-reperfusion injury (IRI), systemic inflammation, and multi-organ failure (5). Despite enormous amounts of research, the underlying pathomechanisms are still poorly understood, hindering a target-oriented therapy.

Hemorrhagic shock is known to cause a mismatch between oxygen supply and demand. The tissue hypoxia that occurs results in pathophysiological disturbances of the cellular machinery. Protein maturation and folding in the endoplasmic reticulum (ER) is a highly energy-dependent cellular process (6). Perturbations in the ER homeostasis result in an impaired ER function and an accumulation of unfolded proteins in the ER lumen-a condition defined as ER stress (7). Consequently, the cell activates specific signaling pathways, which are collectively known as the unfolded protein response (UPR) consisting of three primary branches: Protein kinase RNA-like endoplasmic reticulum kinase (PERK), activating transcription factor 6 (ATF6), and inositol-requiring enzyme 1 (IRE1). Under physiological conditions, binding immunoglobulin protein (BiP), a molecular chaperone and master regulator of ER function, binds to the luminal domains of PERK, ATF6, and IRE1(8). Upon ER stress, BiP dissociates from each stress sensor and facilitates their activation. Whereas PERK and IRE1 undergo oligomerization and trans-autophosphorylation (9), ATF6 is activated by proteolytic cleavage in the Golgi compartment (10). After its translocation to the nucleus, ATF6 promotes the transcription of genes coding for adaptive proteins, such as chaperones, and of X-box binding protein 1 (XBP1) mRNA (11). Before its translation, XBP1 mRNA is spliced by activated IRE1 endoribonuclease (12). XBP1 codes for an active transcription factor, which amplifies the synthesis of components of the ER-associated protein degradation machinery (13). Additionally, the activation of the PERK pathway results in a global attenuation of protein synthesis by phosphorylation of eukaryotic initiation factor 2α (eIF2α) (14). In brief, the UPR is primarily a pro-survival cellular response aiming to restore protein homeostasis in the ER by facilitating protein folding, reducing protein synthesis, and increasing protein degradation. However, if the UPR fails to reestablish protein homeostasis and ER stress persists, cell death may occur (15).

As reported in previous studies, the above-described cellular mechanisms influence the extent of liver damage following hemorrhagic shock and reperfusion (HS/R) (16-18). Furthermore, the UPR mediates the protective effects of stress preconditioning, an established concept to mitigate subsequent IRI. Previous studies have demonstrated that ischemia-reperfusion associated hepatocellular, myocardial, and neuronal cell damage can be alleviated by stress preconditioning (e.g. by remote ischemic preconditioning or lipopolysaccharide pretreatment) (19,20). From a clinical point of view, the therapeutic potential of this method has already been demonstrated by the bench-to-bedside transfer of remote ischemic preconditioning in patients undergoing coronary artery bypass surgery (21,22). Even though IRI and HS/R signaling pathways have been studied for decades, some of the underlying mechanisms remain elusive. However, developing novel therapeutic approaches requires a deeper understanding of the pathophysiology of IRI and HS/R. Based on the above-described findings and our previous results, we hypothesized that ER stress preconditioning alleviates liver damage following HS/R and may thereby reveal a target with potential therapeutic relevance. To investigate this hypothesis and to identify the underlying protective mechanisms, we injected mice with the pharmacological ER stress inducer tunicamycin (TM) and subjected them to HS/R 48 h later.

## Materials and methods

### 

#### Animal care

C57BL/6 mice, 10 weeks of age, were purchased from Charles River Laboratories (Sulzfeld, Germany). Due to the influence of female sex steroids on post-hemorrhagic organ damage, only male mice were included (23). The animals were housed in the animal facility of the University of Heidelberg at a temperature of 21˚C and a 12 h light/dark cycle. The mice had one to two weeks for acclimatization and had access to water and chow *ad libitum*. During acclimatization the animals were housed in group cages and with start of the experiment animals were placed individually. All study protocols were reviewed and approved by the section for agriculture and veterinary services of the Regional Council, Karlsruhe, Germany (35-9185.81/G-65/13).

#### Experimental model

The shock protocol was performed as previously published (17,18). Briefly, anesthesia was induced via inhalation of 4% isoflurane (Abbott Laboratories Ltd.) in an acrylic glass chamber. After loss of righting reflex the animals were placed in a supine position on a heating cushion and anesthesia was maintained by administering ~1.2% isoflurane via a face mask. For temperature control (37.0±0.5˚C) a rectal probe was inserted. Before the bilateral dissection of the groins, 25 µl (~5 mg/kg body weight) of 0.5% Bupivacaine hydrochloride (AstraZeneca Gmbh) was applied for local anesthesia intraincisionally. Subsequently, the femoral arteries were cannulated with a polyethylene tubing, previously flushed with a heparin solution. The right catheter was connected to a blood pressure analyzer (BPA-400, Micro-med Inc.), the left catheter was used to withdraw blood and induce a hemorrhagic shock. The mean arterial pressure (MAP) was maintained for 90 min at 30±5 mmHg. Afterwards, Ringer's solution, three times the shed blood volume, was injected for resuscitation. Subsequently, the catheters were removed, the vessels were ligated, and the skin was closed. After the discontinuation of isoflurane inhalation, the mouse was placed in its cage and observed till emergence.

Mice were randomly assigned to five different groups. HS/R groups were treated as outlined above. Depending on the group assignment mice received either TM (0.75 mg/kg BW in solution, Merck KGaA) or its drug vehicle (DV) dimethyl sulfoxide (DMSO), dissolved in 100 µl Ringer's solution, which was given intraperitoneally 48 h before shock induction. Sham controls (SC) were created for each HS/R group. SC groups received the same treatment as the corresponding HS/R group but did not undergo hemorrhagic shock. For the evaluation of physiologic baseline values, euthanasia was performed under anesthesia without any prior treatment given to the mice. This baseline control (BC) group as well as the HS/R+TM group consisted of six animals whereas all other groups (SC+DV, SC+TM, HS/R+DV) contained three animals. Experimental group size calculation was based on our previous study, which compared mice undergoing HS/R procedure and receiving the drug vehicle (DV) DMSO during reperfusion with mice undergoing HS/R procedure without any pharmaceutical intervention (18). Each group included 6-7 animals. The same comparison was performed for mice undergoing sham procedure with 5 animals per group. Using Mann-Whitney U test we could not find any significant differences in transaminase levels or percentage of cell death areas (HS/R groups). Therefore, we concluded that the applied dosage of our solvent DMSO does not influence our main outcome parameters and decided to limit the number to three animals per control group in the present study.

#### Tissue harvesting and plasma analysis

A period of 14 h after shock induction, anesthesia was induced and maintained via inhalation of 4% isoflurane. After loss of the paw withdrawal reflex and observation of agonal breathing, a laparotomy and thoracotomy were performed. For euthanasia the right heart ventricle was punctured using a heparinized 1-ml syringe. Death was confirmed by observation of cardiac and respiratory arrest. The collected blood was centrifuged (10 and 5 min at 2,000 x g) and 50 µl of the plasma was used to measure aspartate aminotransferase (ASAT) as well as alanine aminotransferase (ALAT) concentrations (Fuji Dri-Chem NX500i; FujiFilm Europe GmbH). Subsequently, the body was flushed with a heparin solution through puncture of the left ventricle. The liver was then harvested and halved. One half was snap-frozen by submerging the sample tubes into liquid nitrogen and the other half was placed in 4% paraformaldehyde (PFA).

#### Histology

After fixation in 4% PFA for at least 24 h the livers were dehydrated using a series of alcohols with increasing concentrations and acetone. Hereafter, the organs were embedded in paraffin. The tissues were then cut into 5 µm sections. For deparaffinization the slides were placed in xylene and afterwards immersed in a series of alcohols with decreasing concentrations for rehydration. The sections were then either processed for immunohistochemistry or stained with hematoxylin and eosin (H&E) using a standard protocol. To quantify liver damage, the H&E-stained liver tissue sections were assessed for dead cells assessed by at least two investigators experienced in analyzing histological slides. We first measured the percentage of vessels and dead cells using ImageJ (Version: 1.51f; Wayne Rasband, National Institutes of Health). In the following, the number of pixels covered by vessels were subtracted from the total pixel amount and the percentage of irreversibly damaged tissue was calculated. Corresponding morphological features were a rupture of the nuclear envelope or chromatin condensation, loss of cell borders with irregular fragmentation and/or washed-out image of cytoplasm (24,25). Since cell swelling per se is a reversible state, swollen cells were not considered as dead cells (24). Six-eight representative visual fields (100 x) per animal of the HS/R+DV and 1-5 representative visual fields per animal of the HS/R+TM group were analyzed. In total, we evaluated 20 representative visual fields for each HS/R group. The varying numbers of analyzed visual fields per animal resulted from the different group sizes. Subsequently, the median percentage of damaged tissue per animal was calculated and used for further statistical analysis.

#### Immunohistochemistry

Immunohistochemical staining was performed as previously published (17,18). In the following, we describe the BiP staining more detailed since that was our standard protocol. Therefore, only the differences to the BiP staining process are mentioned for the other staining procedures.

#### BiP

After deparaffinization and rehydration the tissue sections were immersed in 0.45% hydrogen peroxide for 20 min to block the endogenous peroxidase activity. Heat-induced antigen retrieval was performed by placing the sections for 20 min in a citrate buffer (pH 6.0, 10 mM) set to 100˚C. Following this, the blocking agent, 1.5% donkey serum (#sc-2023, Santa Cruz Biotechnology, Inc.) in phosphate buffered saline (PBS), was applied. The tissue sections were then incubated overnight at 4˚C with the primary antibody, goat anti-BiP (#sc-1050, Santa Cruz Biotechnology, Inc.), at a dilution of 1:50. In the next step, the secondary antibody, donkey anti-goat IgG (#sc-2023, Santa Cruz Biotechnology, Inc.), was administered for 30 min at room temperature, diluted at 1:200. For signal detection, alkaline phosphatase (AP; #AK-5000, Vector Laboratories) was applied and Fast Red was used as chromogen. To stop the reaction, the slides were immersed in distilled water. Finally, hematoxylin was applied for counterstaining and the slides were mounted using an aqueous mountant.

#### ATF6

2.5% horse serum was used as a blocking solution (#MP-5401, Vector Laboratories, Inc.). The tissue sections were incubated overnight at 4˚C with the rabbit anti-ATF6 antibody (#NBP1-77251, Novus Biologicals Europe, Cambridge, Great Britain), diluted at 1:100. As secondary antibody we applied a horse anti-rabbit antibody for 30 min at room temperature, which was supplied as ready-to-use kit and already conjugated with alkaline phosphatase by the manufacturer (#MP-5401, Vector Laboratories, Inc.).

#### pPERK

To block unspecific antibody binding sites, a solution of 5% skim milk and 1% BSA was applied. Afterwards, the primary antibody, rabbit anti-pPERK (#ab192591, Abcam plc.,), incubated overnight at 4˚C at a 1:50 dilution. The slides were then covered with the secondary antibody for 30 min at room temperature, a ready-to-use alkaline phosphatase polymer anti-rabbit reagent (#MP-5401, Vector Laboratories, Inc.).

#### sXBP1

After the blocking procedure with 1.5% donkey serum (#sc-2023, Santa Cruz Biotechnology, Inc.), the slides were incubated overnight at 4˚C with the goat anti-XBP1 antibody (#ab85546, Abcam plc.), diluted at 1:100. Subsequently, the secondary antibody (#sc-2023; Santa Cruz Biotechnology, Inc.), diluted at 1:200 in 1.5% donkey serum, was applied for 30 min at room temperature.

#### Beclin 1

For deparaffinization the slides were placed in xylene before being rehydrated by immersion in 100% isopropanol. Afterwards, the endogenous peroxidase activity was blocked by 1.5% methanol and antigen retrieval was performed as described above. To block unspecific binding sites 2.5% horse serum was applied. Next, the slides were incubated for one hour at room temperature with the primary antibody, rabbit anti-Beclin1 IgG (#NB500-249, Novus Biologicals Europe), at a dilution of 1:400 before the secondary antibody (#MP-5401, Vector Laboratories, Inc.) was applied for 30 min at room temperature.

#### Western blot analysis

The frozen livers were thawed and homogenized using a homogenization buffer (5 mmol/l 3-(N-morpholino) propanesulfonic acid, 1 mmol/l ethylenediaminetetraacetic acid, 0.25 mol/l sucrose, 0.2 mmol/l dithiothreitol, 1 mmol/l ε-aminocaproic acid, 5 mmol/l benzamidine, 0.2 mmol/l phenylmethylsulfonyl fluoride, 0.1% Triton X-100). After centrifugation at 15.400 g for 15 min at 4˚C the protein samples were fractionated by electrophoresis on sodium dodecyl sulfate polyacrylamide gel. Subsequently, the separated proteins were transferred to a polyvinylidene fluoride membrane (#1620177, Bio-Rad Laboratories GmbH). The membranes were then washed with PBS-Tween (0.05%) followed by the saturation with 5% skim milk in PBS-Tween for one hour at room temperature to block non-specific binding sites. Afterwards, the membranes were incubated overnight at 4˚C with the primary antibody, goat anti-BiP (#sc-1050, Santa Cruz Biotechnology, Inc.), diluted at 1:500 in 5% skim milk. Before and after the incubation for one hour at room temperature with the HRP-conjugated secondary antibody, donkey anti-goat IgG (#sc-2020, Santa Cruz Biotechnology, Inc.) diluted at 1:5,000 in 5% skim milk, the membranes were washed with PBS-Tween. Enhanced chemiluminescent substrate was added and the signal was detected using Image Reader LAS-3000 Version 2.0 (Fuji Photo Film). For loading control, the membranes were incubated for two hours at room temperature with rabbit anti-glyceraldehyde 3-phosphate dehydrogenase (GAPDH) antibody (#sc-25778, Santa Cruz Biotechnology, Inc.) diluted at 1:500 in 1% newborn calf serum.

Regarding the quantification of BiP expression, we loaded two reference samples on each plot to allow a comparison of different plots. For the analysis we used the ImageJ (Version: 1.51f; Wayne Rasband, National Institutes of Health). We selected the lanes, plotted them and labelled the peaks. After converting the number of pixels of each lane into percentage we divided the percentage of each sample by the percentage of our reference sample. These steps were performed for BiP as well as for GAPDH. We finally divided the calculated values and received the ratio of BiP to GAPDH expression. Using this approach, the analysis was adjusted to inconsistent GAPDH expressions.

#### Statistical analyses

Statistical analysis was performed using MATLAB R2018b (The MathWorks Inc.). Shapiro-Wilk test showed a non-parametric distribution of the data. Therefore, the Kruskal-Wallis test was used for the comparison of more than two groups, together with Tukey-Kramer post-hoc correction. For the comparison of two groups the Wilcoxon Mann-Whitney test was applied. Data are expressed as median [minimum; maximum]. A p-value below 0.05 was considered statistically significant. We additionally present the Area Under the Curve (AUC) with bootstrapped 95% confidence intervals as effect size. For this we used the MATLAB-based MES toolbox (26). The use of AUC helps to evaluate the strength of an effect (27).

## Results

### 

#### Model evaluation

**Figure 1:**
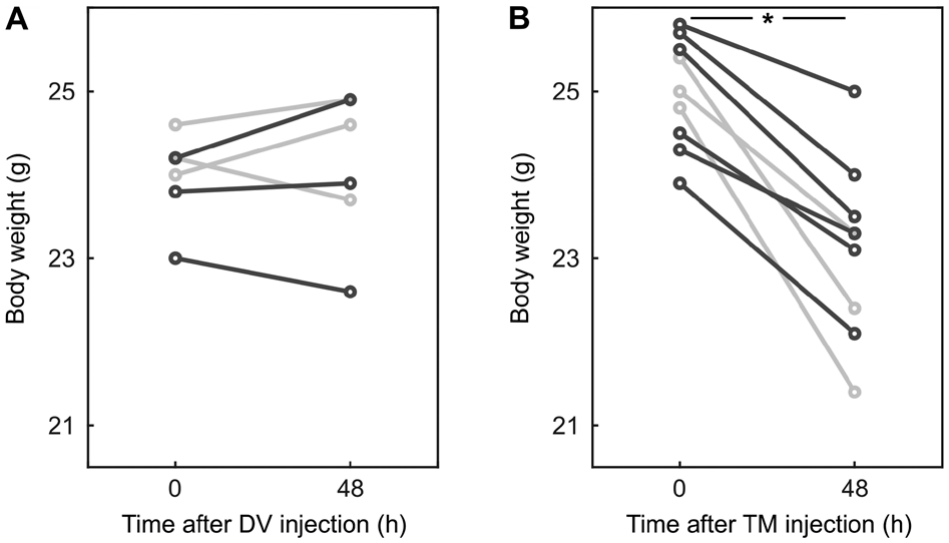
TM injection was followed by body weight loss. Display of body weight in gram (g) at the time of the injection and before the beginning of the surgical procedure, 48 h later. The light gray lines represent animals of the sham control and the dark gray lines animals of the hemorrhagic shock and reperfusion group. (A) The body weight of mice (n=6) receiving the DV at 0 and 48 h. (B) Mice treated with TM injection mice at 0 and 48 h. ^*^P<0.01 (n=9).TM, tunicamycin; DV, drug vehicle.

Fixed pressure-controlled hemorrhagic shock was shown to be a reliable and reproducible model (28). The mice used in this experiment did not differ in age, body weight, or strain from previous studies (17,18). As four animals had to be excluded, e.g. due to death during hemorrhagic shock (n=1), malformations (n=1), or inconsistent shock with more than three peaks above 35 mmHg (n=2), we included 21 animals in our analysis. The mean blood volume to induce and maintain a mean arterial pressure of 30±5 mmHg for 90 min was 0.60 [0.50; 0.65] ml (2.45 [2.10; 2.94] ml per 100 g body weight) and did not differ significantly between the shock groups. Furthermore, there were no significant differences between the groups regarding the body weight at the start of the experiment. However, mice which received TM lost 6.8 [3.1; 13.7]% of their body weight within 48 h (P=0.004; AUC 0.93 [0.78 1]; Fig. 1).

#### TM preconditioning alleviated liver damage

**Figure 2:**
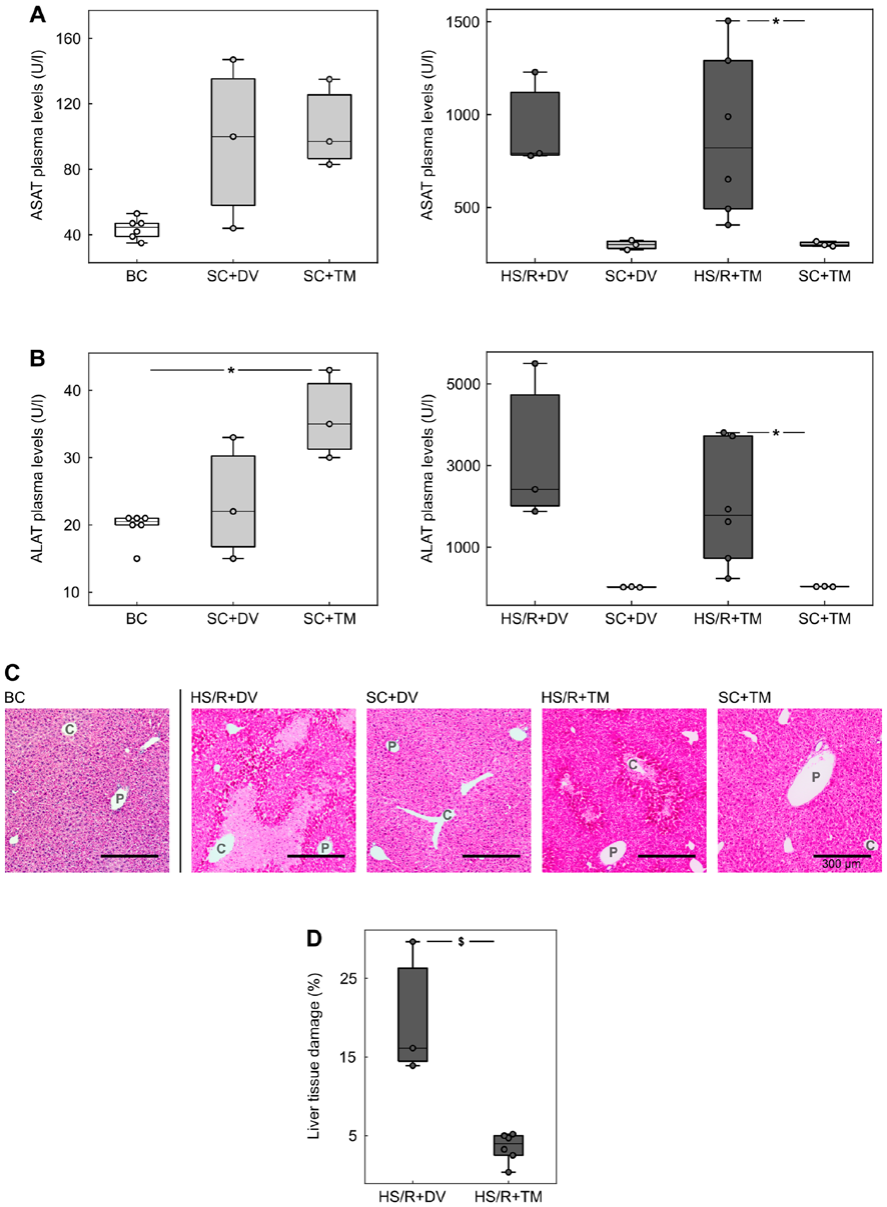
TM preconditioning mitigated liver damage after HS/R. (A) ASAT and (B) ALAT plasma levels in Units per liter (U/l). (C) Representative hematoxylin and eosin stains of liver tissue sections. Scale bar, 300 µm. (D) The quantification of cell death areas after HS/R was performed by analyzing 6-8 representative visual fields (magnification, x100) per animal of the HS/R+DV (n=3) and 1-5 representative visual fields per animal of the HS/R+TM group (n=6). One circle displays the median percentage of damaged tissue of one animal. ^*^P<0.05; ^$^P<0.01. HS/R, hemorrhagic shock and reperfusion; ASAT, Aspartate aminotransferase; ALAT, alanine aminotransferase; DV, drug vehicle; TM, tunicamycin.

To assess the extent of liver damage, plasma concentrations of ASAT and ALAT were measured as their plasma levels correlate with hepatocellular injury (29) (Fig. 2A and B). The comparison of the control groups (BC, SC+DV, SC+TM) showed no significant differences except for the ALAT concentration in SC+TM group (35.0 [30.0; 43.0] U/l), which was significantly higher than in BC (20.5 [15.0; 21.0] U/l, P=0.047; AUC 0 [0 0]). The ASAT/ALAT levels in the shock groups were similar: 1141.5 [312.0; 2510.0] U/l and 1778.0 [235.0; 3805.0] U/l in HS/R+TM group vs. 1084.0 [1058.0; 1958.0] U/l and 2417 [1876.0; 5499.0] U/l in the HS/R+DV group. The comparison of the shock groups with their corresponding sham groups demonstrated more than 10-fold higher transaminases concentrations in the HS/R groups. This difference was significant for TM groups (P=0.024; AUC 0 [0 0]) and non-significant for the DV groups (P=0.1).

Additionally, H&E-staining of liver tissue sections was performed to confirm the results of the plasma measurements (Fig. 2C and D). The evaluation of baseline and sham controls showed no obvious signs of hepatocellular damage. In contrast to this finding, there were cell death areas spreading centrifugally from the central vein in both shock groups. The quantification of these areas revealed that the percentage of damaged liver tissue was significantly lower in the HS/R+TM group (4.0 [0.4; 13.9]%) compared to the HS/R+DV group (16.1 [5.2; 30.0]%; P=0.024). This finding is underlined by an AUC of 0 [0 0].

#### BiP expression was upregulated by TM preconditioning

**Figure 3:**
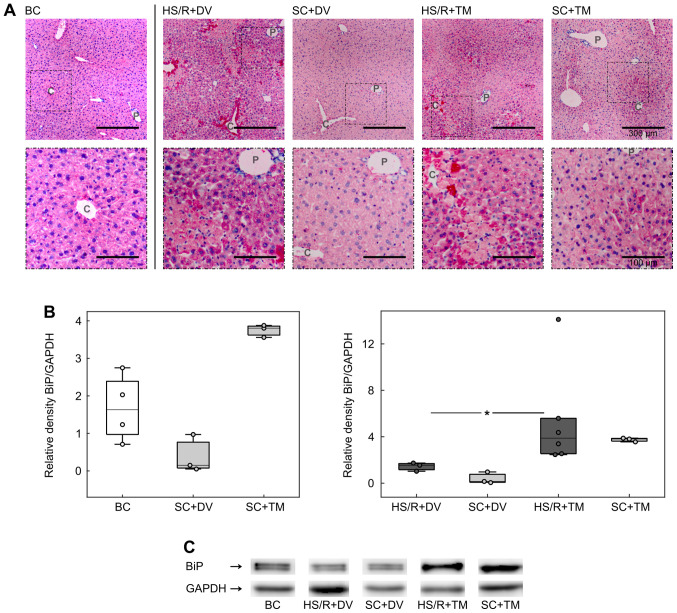
TM preconditioning induces BiP expression. (A) Representative immunostainings of the endoplasmic reticulum stress marker BiP. Scale bar of upper row, 300 µm; Scale bar of lower row, 100 µm. Vessels of the periportal field (P) and central veins (C) are exemplified. (B) Protein expression was quantified by calculating the relative density of BiP to GAPDH. SCs and HS/R+DV included three animals, BC and HS/R+TM consisted of six mice. ^*^P<0.05. BiP, binding immunoglobulin protein; SC, sham control; HS/R, hemorrhagic shock and reperfusion; DV, drug vehicle; TM, tunicamycin; BC, baseline control.

To investigate the influence of TM preconditioning on the ER, we analyzed the expression of BiP, a master regulator of ER function and a known ER stress marker (8,30). Immunohistochemistry displayed a homogeneous staining of the liver sections in BC and SC+DV groups (Fig. 3A). In the HS/R+DV group the vital parenchyma was also homogenously stained but the staining intensity appeared to be higher compared to its corresponding sham group. Furthermore, there were single, intensely stained cells adjacent to the cell death areas. These cells were also seen in the HS/R+TM group. However, the BiP baseline expression pattern in HS/R+TM and SC+TM varied from all other groups. Liver tissue sections of mice, which received TM, displayed an increasing gradient of BiP expression from the periportal field to the central vein.

For the evaluation of BiP expression in whole liver homogenates western blotting was performed (Fig. 3B and 3C). The analysis demonstrated an increased BiP expression in both TM groups. Compared to the HS/R+DV (1.55 [1.03; 1.74]), the HS/R+TM group (3.88 [2.46; 14.11]) showed a significantly higher BiP/GAPDH ratio depicted by P=0.024 and an AUC of 1 [1 1].

#### Topographical changes in the UPR after TM preconditioning

**Figure 4:**
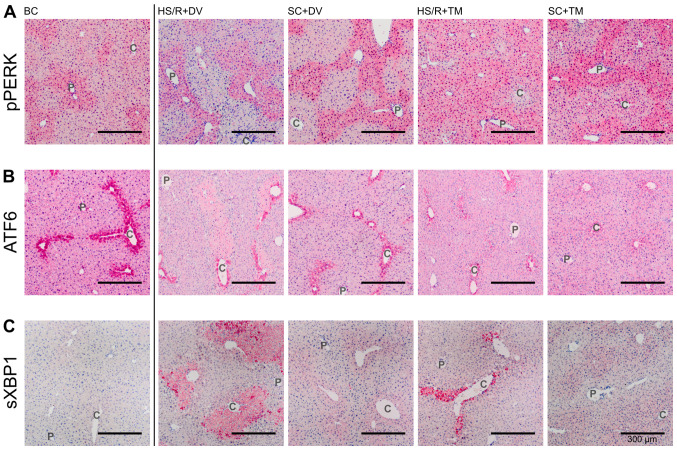
Topographical changes of UPR signaling. Representative stains of the immunohistochemical proof of pPERK, ATF6, and sXBP1. Vessels of the periportal field (P) and central veins (C) are exemplified. Immunohistochemical staining for detection of (A) pPERK, (B) ATF6 and (C) sXBP1 in the different groups. Scale bar, 300 µm. pPERK, phosphorylated protein kinase RNA-like endoplasmic reticulum kinase; ATF6, activating transcription factor 6; sXBP1, spliced Version of X-box binding protein 1; SC, sham control; HS/R, hemorrhagic shock and reperfusion; DV, drug vehicle; TM, tunicamycin; BC, baseline control; UPR, unfolded protein response.

Since PERK undergoes autophosphorylation upon ER stress, phosphorylated PERK (pPERK) indicates its activation (9). The pPERK staining was predominated by an increased staining intensity around the periportal field (Fig. 4A). This pattern was observed in the BC, SC+DV, SC+TM, and HS/R+DV group. However, the difference in the periportal and pericentral staining intensity appeared to be smaller in the SC+TM group compared to the SC+DV group. Liver tissue sections of HS/R+TM group showed a similar pericentral as well as periportal expression level displayed by a homogeneous, intense staining.

ATF6 is constitutively expressed (31). Upon ER stress, its activation is initiated by the dissociation from BiP, followed by proteolytic cleavage in the Golgi compartment (32). The ATF6 expression pattern was characterized by a homogeneous staining of the vital liver parenchyma and one row of intensely stained cells around the central vein (Fig. 4B). In the SC+TM group the pericentral cells were not as intensely stained as in the other groups. In this group, a smooth transition from the intensely stained pericentral area to the remaining liver parenchyma was detected.

Since activated IRE1 splices XBP1 mRNA, measuring spliced XBP1 (sXBP1) is a reliable, indirect method of assessing IRE1 activation (30). In the BC group only a slight, homogeneous expression could be detected (Fig. 4C). The staining pattern in SC+DV was characterized by an increased pericentral intensity that faded out centrifugally. Whereas in HS/R+DV a sharp transition between intensely stained cell death areas and slightly stained vital parenchyma was found the staining pattern in SC+TM and HS/R+TM was similar to SC+DV.

#### TM preconditioning induced pericentral autophagy

**Figure 5:**
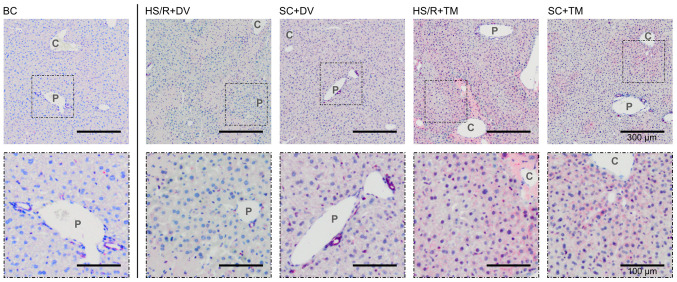
Pericentral Beclin1 upregulation by TM preconditioning. Immunohistochemical stained liver tissue sections for the autophagy marker Beclin1. Vessels of the periportal field (P) and central veins (C) are exemplified. Scale bar of upper row, 300 µm; Scale bar of lower row, 100 µm. SC, sham control; HS/R, hemorrhagic shock and reperfusion; DV, drug vehicle; TM, tunicamycin; BC, baseline control.

Beclin1 is a known marker of autophagy, which is naturally expressed in biliary epithelium (33). In the BC, SC+DV, and HS/R+DV group Beclin1 was scarcely expressed indicated by a weak and partly missing staining of the vital parenchyma (Fig. 5). In contrast to this finding, both TM groups showed an increased Beclin1 expression pericentrally. Similar to the BiP staining pattern an increasing staining intensity from the periportal field to the central vein was observed.

## Discussion

As previously shown by Jian *et al* (16), ER stress plays an important role in liver injury following HS/R. We confirmed this finding in our preceding study: The injection of the ER stress inhibitor tauroursodeoxycholic acid (TUDCA) during reperfusion mitigated hepatocellular damage, whereas the administration of the ER stress inductor TM during reperfusion increased hepatocellular damage (18). In addition, we conducted a detailed timeline investigation of the temporal dynamics of the expression of UPR signaling proteins and liver injury (17). Our analysis revealed a maximum of hepatocellular damage 14 h after shock induction. Since the focus of the present study was on liver damage, we chose to sacrifice mice 14 h after hemorrhagic shock induction.

TM is a pharmaceutical ER stress inducer and acts via an inhibition of N-glycosylation, causing an accumulation of unfolded glycoproteins in the ER (30,34). In accordance with previous studies, the results of the western blot analysis of whole-organ homogenates demonstrated an increased BiP expression following TM pretreatment (35). As prior publications on IRI and HS/R have already demonstrated that an increased expression of BiP is accompanied by an upregulation of pPERK, IRE1, and ATF6 (16,36,37) and as the focus of the present study was also on the topographical distribution of ER stress, we decided to only analyze BiP in whole liver homogenates. We chose BiP since it is a master regulator of ER function. As a member of the heat shock protein 70 family, BiP is a highly conserved molecular chaperone (8). However, it not only facilitates protein folding, but is also an essential component of quality control mechanisms of the secretory pathway and regulates endoluminal calcium concentration (38,39). Additionally, and of the utmost importantance for the present study, BiP is a well-established marker of ER stress as its expression is induced by mal-/unfolded proteins (40,41).

Interestingly, immunohistochemistry showed that BiP was not homogeneously increased but rather focused in the pericentral area. This topographic distribution pattern might be explained by the unique blood supply of the liver, which leads to a decreasing oxygen gradient from the periportal region towards the pericentral area (42). In several studies, Paxian *et al* (43,44) demonstrated the resulting susceptibility to external stressors of pericentral hepatocytes, especially during hemorrhagic shock and the oxidant stress upon reperfusion. Consequently, these cells respond more sensitively to TM than periportal cells, indicated in the present study by the increased pericentral expression of ER stress marker BiP in both TM groups (44). Our finding of a topographic correlation of an upregulated BiP induction with the diminution of hepatocellular damage suggests that BiP has beneficial effects. This assumption is also supported by a recent publication from Bi *et al* (45), which demonstrated that an overexpression of BiP mitigated myocardial IRI. In line with the results of Paxian *et al* (43), the protective effect was mediated by inhibiting an accumulation of reactive oxygen species. Taking our observations and the current literature into account, we conclude that a pre-hemorrhagic BiP induction by TM administration mitigates post-hemorrhagic hepatocellular injury.

Whereas TM injection significantly altered BiP expression, its influence on the topographic patterns of ATF6 and IRE1 was limited. This difference might be based on the degradation of the proteins: The half-life of BiP is approximately 46 h, while ATF6 and IRE1 are degraded with a half-life of about 2 and 3 h, respectively (46-48). Consequently, the effect of TM on their topographic patterns might already have faded away 62 h after the injection. In contrast, PERK signaling, represented by pPERK, was markedly influenced by TM pretreatment. The homogeneous staining intensity in HS/R+TM group suggests an upregulation of the PERK pathway in the intermediary and pericentral zone. Even though PERK can contribute to cell death, we theorize that PERK signaling in the context of IRI and HS/R is primarily protective (49). The pro-survival effect might be mediated by the activation of the antioxidant response element via ATF4 and nuclear factor erythroid 2-related factor 2 (Nrf2), resulting in an upregulation of protective enzymes (50). Since PERK activation thereby promotes beneficial effects, the increased pericentral expression of pPERK may explain the reduction of centrilobular cell death areas in the HS/R+TM group. Leung *et al* (51) recently demonstrated in a murine HS/R model that Nrf2 plays a crucial role in the generation of protective factors induced by stress preconditioning and thus confirmed the importance of the PERK/ATF4/Nrf2 signaling branch.

In addition to its function as an activator of Nrf2, ATF4 is a key signal for ER stress induced autophagy (52). Although autophagy can play dual roles and may promote cell death, multiple studies attribute beneficial effects to the autophagic process during hypoperfusion or ischemia (53-55). Chandrika *et al* (53) demonstrated in the context of renal IRI that ER-stress induced autophagy provides cytoprotection. Yan *et al* (56) induced a subarachnoid hemorrhage in rats and described an autophagy dependent mitigation of early brain injury. Moreover, autophagy was shown to be hepatoprotective during a low-flow state, e.g. caused by septic shock (55). Previous studies reported that for correct autophagosome formation BiP is an obligatory component (57). Our immunohistochemical analysis supports this finding as the expression patterns of BiP and the autophagy marker Beclin1 were similar in the TM groups (33). Furthermore, our results underpin the hypothesis of Zhang *et al* (19), attributing a protective role to BiP dependent autophagy induction in the context of IRI. In the HS/R+TM group, Beclin1 expression was upregulated around the central vein, which indicates an increase in autophagic activity in the pericentral zone. With regard to the diminished liver damage in the HS/R+TM group, the topographic distribution of the cell death areas and Beclin1, the present study underlines the protective role of autophagy and identifies its activation as a beneficial mechanism.

Hepatic injury was analyzed by evaluating H&E-stained liver tissue sections and measuring serum transaminases. We chose to use the umbrella term ‘liver damage’ because of the vague understanding of cell death mechanisms during HS/R. Although necrosis has been postulated as the predominant cell death mechanism, the occurrence of apoptosis and autophagy-related cell death has similarly been reported (58,59). In the present work, we focused on ER stress as an underlying mechanism of the IRI as well as on the impact of ER stress preconditioning on organ damage. We did not investigate the exact modalities of ER stress associated cell death. In contrast to the long-standing assumption that prolonged ER stress only triggers apoptotic cell death, recent studies have demonstrated an ER stress induced caspase-independent cell death mechanism (60-62). Therefore, we suggest taking into account histomorphologic signs of both cell death modalities, when investigating post-hemorrhagic hepatocellular damage.

Interestingly, the results of the methods employed were not totally consistent. The discrepancy in the results of the two methods might be explained by the lengthy half-life of the liver transaminases on one hand and the liver's ability to regenerate on the other hand (63). Li *et al* (64) and Pajaud *et al* (65) determined the enormous regenerative potential of the murine liver as their data shows a completion of hepatocyte proliferation 72 h after two-thirds partial hepatectomy. The upregulation of early liver regeneration was particularly remarkable considering the debilitating, pre-hemorrhagic body weight loss of mice receiving TM. This body weight loss might be explained by the interplay of ER-stress and inflammation since it is known that ATF4 induces interleukin-6, which promotes adipose tissue lipolysis (66,67). Therefore, ER-stress induction by TM may initiate an inflammatory response which, in turn, decreased body weight.

Since we focused on a fixed point in time, we can only speculate whether TM preconditioning lowered organ damage or if it merely accelerated liver regeneration. Previous studies focusing on IRI or partial hepatectomy (PH) demonstrated the enormous potential of the liver to regenerate (64,65,68). Furthermore, it has been shown that liver regeneration can be promoted through heat-shock proteins (HSPs) (69). As HSPs are upregulated through ER stress, we assume that TM preconditioning mediates its beneficial effects by accelerating liver regeneration via induction of HSPs, e.g. BiP (8,70,71). However, to fully elucidate the temporal dynamics, a detailed time trial is needed. This trial should also include the analysis of transaminase and protein levels 48 h after TM injection. An upregulation of HSP expression just prior to hemorrhagic shock induction could support our above-mentioned assumption.

Since TM is not soluble in aqueous solution at pH 7.4, the manufacturer recommends using DMSO as a solvent. Since DMSO increases serum transaminases, its influence should also be considered when evaluating the results of the histological and laboratory analysis as it could be another explanation for the aforementioned difference (72). However, the dosage applied in the present study was more than 100-fold below its median lethal dose, and in our previous studies we did not detect considerable differences comparing DV groups with mice undergoing sham or HS/R procedure without any drug injection (18,73). Consequently, we assigned only three animals to each control group as we did not expect to augment scientific knowledge by including more mice and might thereby avoid raising ethical issues.

Furthermore, Beclin1 detection is no absolute criteria for determining autophagic status even though it is an established marker of autophagy onset (33,74) Complementary to our analyses, it would be worth evaluating the expression of e.g. p62 or microtubule associated protein 1 light-chain 3 as these proteins are required for the formation of ubiquitinated protein aggregates and their delivery to the autophagy system (74). Detecting these proteins could confirm our conclusion and enable a deeper analysis of the pericentral autophagic processes. Nevertheless, looking at our data, there are further indicators of the induction of autophagy by TM preconditioning in addition to the increased Beclin1 expression. Furthermore, PERK activation was enhanced around the central vein in HS/R+TM group compared to all other groups. One target of the PERK pathway is ATF4, a key signal for autophagy induced by ER-stress (52). Regarding the concomitant upregulation of BiP, which is an obligatory component of autophagy, these immunohistochemical findings support the assumption of a pericentral autophagy induction by TM preconditioning (57). Furthermore, we performed an immunohistochemical proof of CCAAT/Enhancer Binding Protein Homologous Protein (CHOP; data not shown), which is a target gene of ATF4 and has been shown to promote the transcription of several autophagy genes (49,75). In addition, an increased CHOP expression downregulates protein B-Cell Lymphoma 2 (Bcl-2) (76). As Bcl-2 inhibits Beclin1-dependent autophagy, its downregulation facilitates autophagy induction (77). In the present study, the immunohistochemical analysis of liver tissue sections demonstrated an increased CHOP expression around the central vein in TM groups. As this finding suggests an upregulation of autophagy genes and a suppression of autophagy inhibition, it confirmed the above-mentioned results and supports the hypothesis of a pericentral autophagy induction by TM preconditioning. To further elucidate the importance of this finding, a selective post-hemorrhagic autophagy induction would be useful. Since TM influences BiP expression as well autophagy, this approach would help to differentiate the impact of these two mechanisms and highlight their clinical significance (41,78).

A comparison of BiP western blot and immunohistochemistry reveals the strengths and weaknesses of both methods. Since the semi quantitative analysis of whole liver homogenates did not contain any information about the topographic distribution of BiP, we also performed an immunohistochemical proof of BiP. Unexpectedly, the results of the two methods did not completely overlap. Our evaluation of the immunohistochemical staining suggested the highest BiP expression was in the HS/R+DV group. In contrast to this finding, the western blot analysis showed the highest BiP expression in the TM groups. The strong pericentral upregulation of BiP in TM groups may have outweighed the lower expression in the remaining zones, whereas in the HS/R+DV group the BiP expression in the vital liver parenchyma was not enough to compensate for the cell death areas. To backup this assumption, for the future we propose microdissecting the individual liver zones followed by western blot or PCR analyses. This approach makes possible the detection of topographical changes and their simultaneous quantification.

In the present study, we confirmed previous results reporting a significant role for the UPR in IRI. In addition, we demonstrated that the injection of the ER stress inducer tunicamycin mitigates post-hemorrhagic hepatocellular injury. By analyzing topographic expression patterns, we identified an upregulated BiP expression and a concomitant autophagy induction as potential beneficial mechanisms. In conclusion, ER stress preconditioning alleviates post-hemorrhagic liver damage and may lead to novel therapeutic targets.

## Data Availability

The datasets used and/or analyzed during the current study are available from the corresponding author on reasonable request.
